# The Optimal Radiotherapy Strategy for Patients With Small Cell Lung Cancer and Brain Metastasis: A Retrospective Analysis

**DOI:** 10.1111/cns.70102

**Published:** 2024-11-05

**Authors:** Qian Zeng, Xianjing Chu, Gang Xiao, Jing Zhang, Yingying Zhang, Bin Long, Lei Yang, Zhaohua Tan, Rongrong Zhou

**Affiliations:** ^1^ Department of Oncology, Xiangya Hospital Central South University Changsha China; ^2^ Department of Oncology Xiangya Boai Hospital Changsha China; ^3^ Xiangya Lung Cancer Center, Xiangya Hospital Central South University Changsha China; ^4^ National Clinical Research Center for Geriatric Disorders, Xiangya Hospital Central South University Changsha China

**Keywords:** brain metastases, brain radiotherapy, extensive‐stage small cell lung cancer, immune checkpoint inhibitors

## Abstract

**Background:**

Extensive‐stage small cell lung cancer (ES‐SCLC) is a notoriously aggressive malignancy frequently associated with brain metastases (BMs), presenting substantial therapeutic challenges. This study delves into the effectiveness of immunotherapy combined with diverse radiotherapy, especially the influence of brain radiotherapy (BRT) on survival outcomes in the immunotherapy era.

**Methods:**

ES‐SCLC patients treated at Xiangya Hospital and Xiangya Boai Hospital from February 2020 to June 2024 were retrospectively included. The study focused on patients receiving immune checkpoint inhibitors (ICIs). Metrics included overall survival (OS) and progression‐free survival (PFS), employing univariate and multivariate Cox regression models for statistical analysis.

**Results:**

A total of 393 patients with ES‐SCLC who received ICIs were included in the study. Within the entire cohort, the presence of baseline BMs did not statistically affect OS or PFS. However, thoracic radiotherapy (TRT) was identified as a favorable prognostic factor for both OS and PFS. BRT demonstrated a beneficial effect on OS across both the general cohort and the baseline_BMs subgroup. In patients from the baseline_BMs subgroup who had previously undergone TRT, ICIs plus BRT did not significantly improve OS compared to ICIs alone. Conversely, for patients who had not received prior TRT, adding BRT to ICIs significantly enhanced OS. Among the patients who underwent BRT, 71 received whole brain radiotherapy (WBRT) while 19 opted for stereotactic radiosurgery (SRS). No significant differences in OS and PFS were observed between the SRS and WBRT modalities. The sequence of ICIs relative to BRT was found to influence PFS adversely. Administering BRT before ICIs (RT‐ICI) was associated with worse PFS compared to administering ICIs followed by BRT (ICI‐RT). Additionally, no significant differences in OS and PFS were noted among the three subgroups defined by varying intervals between ICIs and BRT. For patients without baseline BMs, TRT and prophylactic cranial irradiation were associated with delayed onset of brain metastases.

**Conclusions:**

Our study underscores the importance of optimizing treatment strategies and considering the timing and integration of radiotherapy and immunotherapy to improve outcomes for patients with ES‐SCLC, particularly those at risk of or presenting with BMs.

## Graphical abstract

In a cohort of patients with extensive‐stage small cell lung cancer undergoing immunotherapy, this study explored the role of radiotherapy and delve into brain metastases subgroup. Brain and thoracic radiotherapy were identified as favorable prognostic factors for survival in those with brain metastases. Moreover, prophylactic cranial irradiation and thoracic radiotherapy delayed intracranial progression in those initially without brain metastases. These findings underscore the pivotal role of local therapies in the management of extensive‐stage small cell lung cancer during the immunotherapy era.

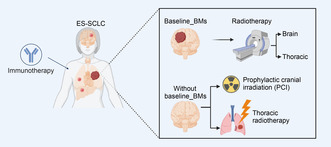

## 1 Introduction

Small cell lung cancer (SCLC), a highly aggressive neuroendocrine malignancy, is characterized by rapid proliferation and a high growth fraction, accounting for 13% of lung cancer cases [1, 2]. Notably, two‐thirds of patients present with distant metastases at diagnosis, a stage often referred to as extensive‐stage (ES)‐SCLC [3, 4, 5]. The prognosis for ES‐SCLC is dismal, with median overall survival hovering around 10 months and 5‐year survival rates stagnating at approximately 6%–7% [6]. Although ES‐SCLC is sensitive to chemoradiotherapy, it is highly prone to relapse and has an extremely poor prognosis [7, 8]. However, the advent of immunotherapy has marked a significant milestone in ES‐SCLC treatment. Clinical trials such as CASPIAN, IMpower133, ASTRUM‐005, and CAPSTONE have demonstrated that anti‐PD‐L1 blockades (atezolizumab, durvalumab, and adebrelimab) and the anti‐PD‐1 blockade serplulimab significantly prolong overall survival (OS), establishing them as the standard first‐line treatment for ES‐SCLC [9, 10, 11, 12]. Nevertheless, many obstacles should be overcome to improve the prognosis of patients with ES‐SCLC.

Approximately half of all patients diagnosed with SCLC will develop BM during their disease course, a complication that is both severe and life‐threatening. The overall survival for these patients is less than 5 months [13]. Even though studies reveal that immunotherapy improves OS irrespective of brain metastasis (BM) presence, a recent retrospective study found that these treatments neither delay median time to brain progression nor mitigate the risk of intracranial metastasis in patients without baseline BMs [14]. Managing brain metastases remains challenging. Standard treatment strategies typically involve local therapies such as brain radiotherapy (BRT), subdivided into stereotactic radiosurgery (SRS) and whole brain radiotherapy (WBRT). In recent years, combining immunotherapy with BRT has emerged as a promising approach for managing BMs. This is due to the dual benefits of immune checkpoint inhibitors (ICIs) and the synergy between brain radiotherapy and ICIs [15]. Firstly, ICIs have been confirmed as an effective option for both extracranial and intracranial lesions [16]. ICIs can cross the blood–brain barrier (BBB) and facilitate T‐cell infiltration into brain tissue, which overcomes the limited penetration of BBB [17, 18]. Secondly, BRT can induce abscopal effects, enhancing the systemic response to immunotherapy [19, 20]. However, high‐quality evidence on the efficacy of BRT for ES‐SCLC in the context of immunotherapy remains scarce. Furthermore, the indications, modalities, timing intervals, and sequencing of BRT with ICIs, remain subjects of debate.

Recurrence of intrapulmonary lesions significantly contributes to treatment failure. Moreover, evidence regarding the combined efficacy of thoracic radiotherapy (TRT) and immunotherapy in treating ES‐SCLC with BM is also notably scarce. Our retrospective study aimed to investigate the effects of radiotherapy in patients with ES‐SCLC and BMs undergoing immunotherapy to identify optimal treatment strategies for this patient cohort and to explore their long‐term survival outcomes.

## 2 Method

### 2.1 Patient Selection and Data Collection

The study participants were patients with ES‐SCLC treated at the Xiangya Hospital of Central South University or Xiangya Boai Hospital between February 2020 and June 2024. The following inclusion criteria were applied: pathologically confirmed ES‐SCLC; complete examination results including laboratory and imaging data, treatment records, and follow‐up results; treated with an anti‐PD‐L1/PD‐1 blockade; and age ≥ 18 years. Patients were excluded if their treatment records were incomplete or if their follow‐up time was less than 1 month. The PD‐1/PD‐L1 inhibitors administered to the patients included Serplulimab, Atezolizumab, Durvalumab, Toripalimab, Tislelizumab, Sintilimab, Adebrelimab, Pembrolizumab, Camrelizumab, Envafolimab, Nivolumab, and Penpulimab. All eligible patients were reviewed and confirmed by the corresponding authors.

Demographic and clinical data were collected, including sex, age at initial diagnosis, history of smoking and alcohol consumption, ECOG PS, extracranial metastases, presence and number of BMs, diameter of the largest BM, clinical symptoms, treatment regimen (radiotherapy, chemotherapy, and immunotherapy), date of death and tumor progression, and adverse events. Patients who received immunotherapy before, during, or within 1 month after radiotherapy were included in the BRT group. Data were collected through to June 2024. The study was approved by the ethics committee of Xiangya Hospital, Central South University (No. 202303038) and performed in accordance with the Declaration of Helsinki.

### 2.2 Endpoints

OS was defined as the time from the start of treatment to the date of death from any cause. PFS was defined as the time from the start of treatment to disease progression, or death from any cause. Intracranial progression‐free survival (iPFS) was defined as the time from the start of treatment to radiologically confirmed intracranial progression or death. Efficacy of treatment was evaluated using Response Evaluation Criteria in Solid Tumors version 1.1. Primary lung and metastatic lesions were evaluated after each two cycles of treatment and the curative effects by CT, PET‐CT, bone scans, or MRI.

### 2.3 Statistical Analysis

In order to objectively compare the prognosis of patients in different treatment groups, baseline characteristics of patients with different treatment groups need to be compared. Categorical variables were tested by chi‐squared test or Fisher's exact test. For continuous variables, if the data exhibited a normal distribution, they were analyzed using the unpaired Student's *t*‐test. If the data, as determined by the Shapiro–Wilk test, did not exhibit a normal distribution, the Mann–Whitney *U* test was employed as the nonparametric alternative. The Kaplan–Meier method was used to estimate PFS, iPFS, and OS, and a log‐rank test was used to compare survival curves. Univariate and multivariate analyses were performed using a Cox proportional risk model to determine the correlation between OS, PFS, iPFS, and important clinical factors. All statistical analyses were conducted by SPSS 27.0 software and R software (4.3.2). *p* < 0.05 was considered statistically significant.

## 3 Results

### 3.1 Patient Characteristics

From February 2020 to June 2024, our study identified 393 ES‐SCLC patients who underwent ICI combined with chemotherapy at Xiangya Hospital and Xiangya Boai Hospital (refer to Table 1). The cohort had a median follow‐up of 13.1 months (ranging from 1 to 66 months). Table 1 delineates the clinical characteristics of the patients. The median ages were 60.6 ± 8.47 years for patients with baseline BMs and 61.7 ± 7.57 years for those without. There were no significant differences in age, sex, smoking history, ECOG‐PS, or type of ICIs used. A notable statistical difference was observed in the location of other distant metastases. Specifically, 117 patients without initial BMs had other metastases (refers to pleural or pericardial metastases), compared to only 13 with initial BMs. We posit that the nature of the metastasis (direct infiltration for pleural and pericardial) may not influence subsequent comparisons. A total of 169 patients developed BMs: 100 presented with baseline BMs and 69 exhibited progression posttreatment (Table S1 for detailed information on BMs). The distribution of variables such as ICI type, symptomatic BMs, BRT, and PCI was evenly balanced across the two groups. The posttreatment progression group may present with larger BM lesions and a greater number of BMs. Of these with BMs, 96 patients underwent BRT—53 at baseline and 43 posttreatment progression. The BRT modalities employed included SRS, WBRT, and combinations thereof. Treatment doses varied: SRS typically involved 14–36 Gy in 1–5 fractions (2–6 Gy each), WBRT was usually 30–54 Gy in 10–30 fractions (1.8–4 Gy each), and PCI was administered at a total of 25 Gy over 10 fractions (2.5 Gy each).

**TABLE 1:** Patient characteristics with baseline BMs and without BMs.

	Total cohorts (*N* = 393)	Baseline BMs (*N* = 100)	Without baseline BMs (*N* = 293)	*p* *
Age	61.4 ± 7.81	60.6 ± 8.47	61.7 ± 7.57	0.226
< 65	238 (60.6%)	64 (64.0%)	174 (59.4%)	0.486
Sex, *n* (%)				
Female	42 (10.7%)	9 (9.0%)	33 (11.3%)	0.656
Male	351 (89.3%)	91 (91.0%)	260 (88.7%)	
Smoking, *n* (%)				
Never	76 (19.3%)	19 (19.0%)	57 (19.5%)	1.000
Former/current	317 (80.7%)	81 (81.0%)	236 (80.5%)	
Drinking, *n* (%)				
Never	224 (57.0%)	66 (66.0%)	158 (53.9%)	0.047
Former/current	169 (43.0%)	34 (34.0%)	135 (46.1%)	
ECOG‐PS, *n* (%)				
0–1	218 (55.5%)	60 (60.0%)	158 (53.9%)	0.348
≥ 2	175 (44.5%)	40 (40.0%)	135 (46.1%)	
Baseline metastases, *n* (%)				
Intrapulmonary	82 (20.9%)	16 (16.0%)	66 (22.5%)	0.213
Liver	90 (22.9%)	21 (21.0%)	69 (23.5%)	0.699
Bone	138 (35.1%)	27 (27.0%)	111 (37.9%)	0.065
Adrenal gland	68 (17.3%)	18 (18.0%)	50 (17.1%)	0.952
Others^a^	130 (33.1%)	13 (13.0%)	117 (39.9%)	< 0.001
ICIs, *n* (%)				
PD‐1	204 (51.9%)	50 (50.0%)	154 (52.7%)	0.744
PD‐L1	189 (48.1%)	50 (50.0%)	139 (47.3%)	

Abbreviations: BMs, brain metastases; ICIs, immune checkpoint inhibitors.

^a^ The category “other” in baseline metastases refers to pleural, and pericardial metastases.

* The *p* value is the comparison between the baseline BM and without baseline BM.

### 3.2 Survival of Entire Cohort

**FIGURE 1:**
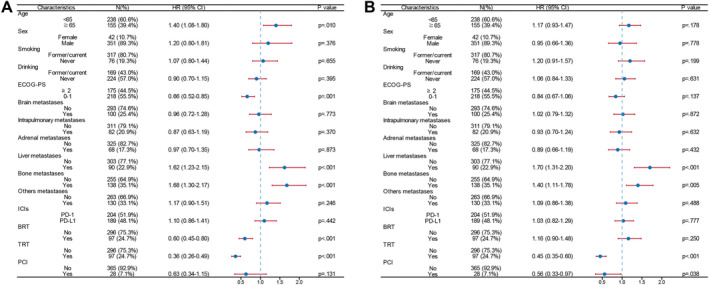
The survival analysis for the entire cohort of patients with extensive‐stage small cell lung cancer. (A) A forest plot illustrating the overall survival. (B) A forest plot presenting systemic progression‐free survival.

As of the data cutoff point, 252 patients (64.1%) experienced mortality. Survival risk factors were examined using Cox proportional hazard models (see Figure 1). This analysis identified six factors significantly correlating with OS: presence of baseline liver or bone metastases, age, BRT, TRT, ECOG‐PS. Notably, ECOG‐PS ≤ 1, BRT, and TRT were identified as good prognostic indicators (hazard ratio [HR] < 1) for OS (refer to Figure 1A). Concerning PFS, significant associations were found with liver and bone metastases, PCI, and TRT. Of these, TRT and PCI were determined as good prognostic factors (HR < 1) for PFS (see Figure 1B). In this study, 100 patients presented with baseline BMs, while 293 patients did not exhibit baseline BMs. A comparative analysis of these groups revealed no significant disparity in median OS and PFS (*p* = 0.773, *p* = 0.872). This suggests that in the era of immunotherapy, baseline BMs may not be an adverse prognostic indicator. Despite the lack of improvement in systemic PFS, BRT significantly extends OS, offering a viable treatment strategy for ES‐SCLC in the era of immunotherapy. TRT not only enhances OS but also PFS, underscoring the significance of local treatment in managing ES‐SCLC.

### 3.3 Subgroup Analysis of Patients With Baseline BMs

After observing the positive response of patients with baseline BMs to ICIs, we sought to explore the optimal treatment for them. We conducted a Cox regression analysis on 100 patients (refer to Table 2). The results indicated that age, ECOG‐PS, receipt of BRT, and TRT were independent prognostic factors for OS. For systemic PFS, sex, age, and TRT emerged as independent prognostic factors. TRT served as a protective factor for both systemic PFS and OS, underscoring the importance of treating the primary lesion with radiotherapy, even when BMs are present. Both BRT and TRT improved OS in patients with BMs, highlighting the importance of incorporating radiotherapy into treatment regimens for SCLC patients, especially in the era of immunotherapy. Although patients treated with PD‐L1 inhibitors appeared to have a poorer prognosis compared to those treated with PD‐1 inhibitors, this difference was not statistically significant.

**TABLE 2:** Analysis of prognostic factors using Cox proportional hazard in patients with baseline BMs.

Variable		*N* (%)	OS		PFS	
			HR (univariable)	HR (multivariable)	HR (univariable)	HR (multivariable)
Age	< 65	62 (62.0%)				
	≥ 65	38 (38.0%)	2.25 (1.36–3.72, *p* = 0.002)	2.26 (1.28–4.00, *p* = 0.005)	1.56 (0.99–2.47, *p* = 0.056)	1.65 (1.00–2.71, *p* = 0.049)
Sex	Female	9 (9.0%)				
	Male	91 (91.0%)	1.55 (0.62–3.87, *p* = 0.346)		0.56 (0.28–1.13, *p* = 0.107)	0.27 (0.10–0.70, *p* = 0.007)
Smoking	F/C	81 (81.0%)				
	Never	19 (19.0%)	1.37 (0.76–2.49, *p* = 0.299)		1.60 (0.95–2.71, *p* = 0.079)	1.08 (0.54–2.14, *p* = 0.832)
Drinking	F/C	35 (35.0%)				
	Never	65 (65.0%)	0.68 (0.41–1.12, *p* = 0.132)	0.66 (0.37–1.17, *p* = 0.155)	0.84 (0.53–1.34, *p* = 0.471)	
ECOG PS	> 2	40 (40.0%)				
	0–1	60 (60.0%)	0.37 (0.22–0.62, *p* < 0.001)	0.38 (0.22–0.66, *p* = 0.001)	0.70 (0.45–1.09, *p* = 0.116)	0.64 (0.40–1.03, *p* = 0.068)
Number of BMs	1 to 4	63 (63.0%)				
	4 to 10	22 (22.0%)	1.75 (0.98–3.13, *p* = 0.061)	1.55 (0.80–2.98, *p* = 0.192)	1.26 (0.74–2.14, *p* = 0.394)	1.21 (0.68–2.13, *p* = 0.518)
	> 10	13 (13.0%)	2.00 (0.94–4.26, *p* = 0.071)	1.65 (0.73–3.70, *p* = 0.227)	1.12 (0.58–2.16, *p* = 0.745)	1.15 (0.55–2.39, *p* = 0.713)
Size of BMs	< 10 mm	48 (48.0%)				
	≥ 10 mm	50 (50.0%)	0.87 (0.53–1.43, *p* = 0.584)	0.69 (0.40–1.17, *p* = 0.167)	0.81 (0.52–1.25, *p* = 0.337)	0.78 (0.48–1.25, *p* = 0.293)
Symptomatic BMs	No	77 (77.0%)				
	Yes	23 (23.0%)	1.06 (0.62–1.81, *p* = 0.841)		1.02 (0.62–1.69, *p* = 0.926)	
Liver metastasis	No	75 (75.0%)				
	Yes	25 (25.0%)	1.50 (0.86–2.61, *p* = 0.153)	1.43 (0.77–2.67, *p* = 0.260)	1.27 (0.77–2.10, *p* = 0.352)	
Bone metastasis	No	70 (70.0%)				
	Yes	30 (30.0%)	1.42 (0.83–2.44, *p* = 0.201)		1.31 (0.81–2.11, *p* = 0.272)	
Intrapulmonary metastasis	No	83 (83.0%)				
	Yes	17 (17.0%)	1.72 (0.91–3.25, *p* = 0.096)	0.81 (0.38–1.73, *p* = 0.582)	1.59 (0.88–2.88, *p* = 0.125)	1.09 (0.57–2.10, *p* = 0.792)
Adrenal gland metastasis	No	80 (80.0%)				
	Yes	20 (20.0%)	1.00 (0.55–1.82, *p* = 0.995)		0.87 (0.51–1.49, *p* = 0.623)	
Others metastasis	No	86 (86.0%)				
	Yes	14 (14.0%)	0.85 (0.40–1.80, *p* = 0.673)		0.95 (0.51–1.77, *p* = 0.878)	
ICIs	PD‐1	50 (50.0%)				
	PD‐L1	50 (50.0%)	1.02 (0.62–1.67, *p* = 0.944)		1.00 (0.65–1.55, *p* = 0.992)	
TRT	No	81 (81.0%)				
	Yes	19 (19.0%)	0.25 (0.11–0.56, *p* < 0.001)	0.32 (0.14–0.74, *p* = 0.008)	0.34 (0.18–0.62, *p* < 0.001)	0.33 (0.17–0.64, *p* = 0.001)
BRT	No	47 (47.0%)				
	Yes	53 (53.0%)	0.47 (0.28–0.76, *p* = 0.002)	0.58 (0.33–1.01, *p* = 0.055)	1.06 (0.68–1.65, *p* = 0.805)	

Abbreviations: BMs, brain metastases; BRT, brain radiotherapy; F/C, former/current; ICIs, immune checkpoint inhibitors; TRT, thoracic radiotherapy; Other metastases refers to pleural, and pericardial metastases.

A subgroup analysis was performed on patients with baseline BMs to identify the population that benefits from BRT (as shown in Figure S1). The analysis revealed that for patients aged 65 and older, male, smokers, nondrinkers, with an ECOG‐PS of 2 or higher, without liver or bone metastases, and with limited asymptomatic brain lesions (1–4 lesions, < 10 mm in size), ICI + BRT significantly improved OS compared to ICI. For patients who had previously undergone TRT, no significant OS improvement was observed with the addition of BRT compared to ICI, suggesting that BRT could be deferred in this group. Conversely, in patients with baseline BMs who had not received TRT, BRT significantly improved their prognosis, underscoring the vital role of localized therapy.

### 3.4 Treatment Modality of BRT

**FIGURE 2:**
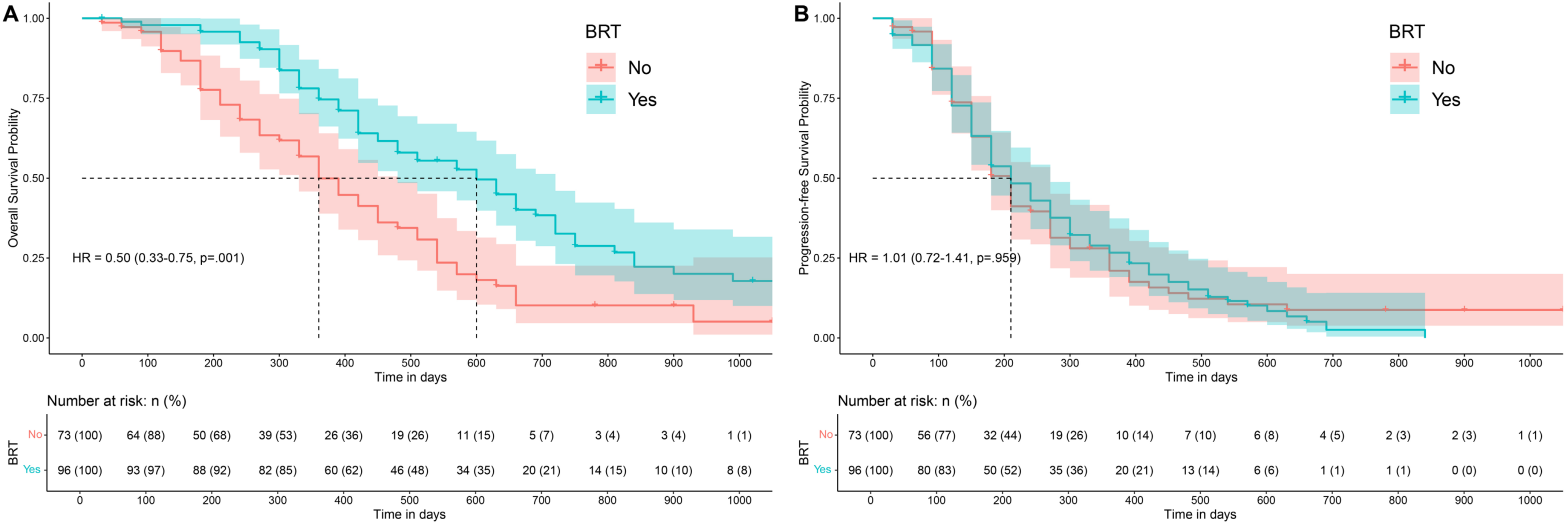
Kaplan‐Meier survival curves for patients with brain metastases who underwent brain radiotherapy. (A) Brain radiotherapy significantly improves OS, evidenced by a hazard ratio (HR) of 0.50 (95% CI: 0.30‐0.75). (B) Brain radiotherapy shows no significant effect on systemic PFS, with a HR of 1.01 (95% CI: 0.72‐1.41).

**FIGURE 3:**
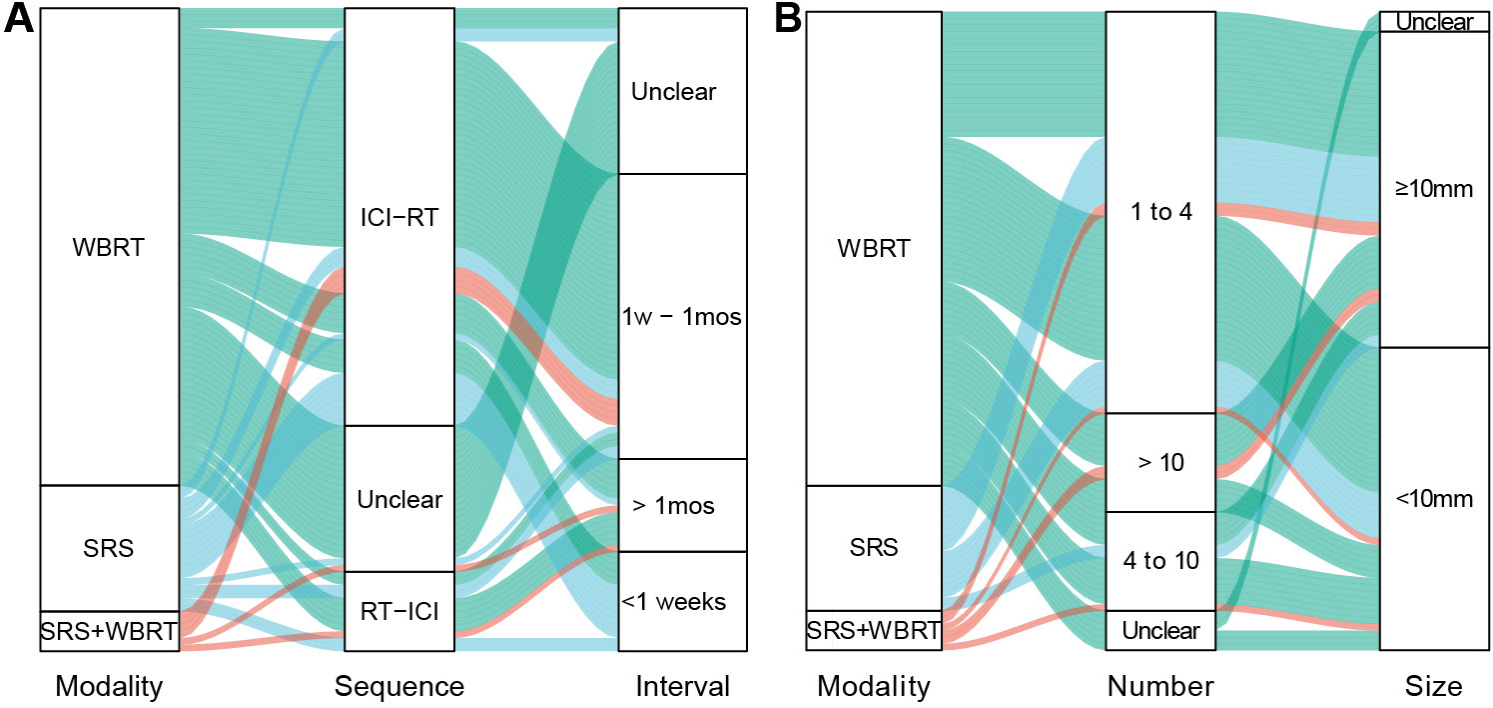
Sankey diagram illustrating the distribution of patient characteristics. Rectangles represent patient subtypes, with the length indicating the proportion of each subtype. Green, blue, and red lines denote patient selections of WBRT, SRS, and combined SRS+WBRT, respectively. (A) The brain radiotherapy strategies, categorized into modality, the sequence of ICI and brain radiotherapy (ICI‐RT indicates ICI prior to brain radiotherapy, RT‐ICI indicates ICI following brain radiotherapy), and the interval between ICI and brain radiotherapy. (B) The clinical characteristics of the patients, segmented by radiotherapy modality, the number of brain metastases, and the diameter of the largest lesion.

Given the crucial role of BRT in treating baseline BMs, we aimed to investigate the factors influencing the efficacy of ICI combined with BRT in BMs (consisting of baseline and progression). The median OS and PFS for patients receiving BRT were 16.3 and 7.3 months, compared to 11.9 and 6.8 months for those not receiving BRT (Figure 2A,B). We firstly delved into whether this outcome was influenced by the BRT design. The Sankey diagram illustrates the subgroups of patients who received BRT, detailing the modalities of radiotherapy, the sequence of radiotherapy and immunotherapy, and the intervals (see Figure 3A). The predominant combination strategy involved administering immunotherapy before BRT (ICI‐RT), with 63 patients adhering to this regimen. Among these, 43 patients had an interval of 1 week to 1 month, whereas 15 patients had intervals of < 1 week. Table 3 details the effects of various BRT modalities such as SRS, WBRT, and their combinations on OS and systemic PFS in patients with BMs. The data reveal no statistical differences in OS and PFS across these BRT modalities. While the sequence of immunotherapy and BRT does not influence OS, starting with BRT prior to immunotherapy (RT‐ICI) correlates with poorer systemic PFS. This underscores the significance of timely immunotherapy in managing SCLC patients with BMs. Moreover, varying the intervals between immunotherapy and BRT has no significant impact on either OS or PFS.

**TABLE 3:** Analysis of survival effects of different BRT designs.

Variable		*N* (%)	OS		PFS	
			HR (univariable)	HR (multivariable)	HR (univariable)	HR (multivariable)
Modality	SRS	19 (19.6%)				
	SRS + WBRT	6 (6.2%)	1.33 (0.46–3.80, *p* = 0.598)		1.19 (0.47–3.01, *p* = 0.721)	
	WBRT	72 (74.2%)	1.08 (0.53–2.22, *p* = 0.823)		0.71 (0.41–1.21, *p* = 0.207)	
Sequence	ICI‐RT	63 (64.9%)				
	RT‐ICI	12 (12.4%)	0.98 (0.44–2.21, *p* = 0.965)		2.04 (1.08–3.88, *p* = 0.029)	2.32 (1.16–4.66, *p* = 0.018)
Interval	< 1 weeks	15 (15.5%)				
	> 1 month	14 (14.4%)	0.83 (0.35–2.01, *p* = 0.682)		0.83 (0.38–1.84, *p* = 0.652)	
	1 week–1 month	43 (44.3%)	0.94 (0.45–1.97, *p* = 0.872)		1.01 (0.54–1.90, *p* = 0.975)	

Abbreviations: ICI, Immune checkpoint inhibitors; RT, radiotherapy; SRS, stereotactic radiosurgery; WBRT, Whole brain radiotherapy.

Although SRS and WBRT show no differences in prognosis, many clinicians have noted that WBRT may significantly impair cognitive functions. To further explore this, we utilized a Sankey diagram to visualize the clinical characteristics of patients choosing SRS or WBRT (Figure 3B). Among the 71 patients opting for WBRT, 41 (57.7%) presented with only 1–4 BMs, and 31 (43.6%) had lesions < 10 mm. In contrast, of the 19 patients selecting SRS, 17 (89.4%) had 1–4 BMs, and 12 (63.1%) had lesions ≥ 10 mm. Despite the limited sample size, these findings suggest that SRS may be more suitable for patients with fewer BMs (fewer than four lesions).

### 3.5 Subgroup Analysis of Patients Without Baseline BMs

**FIGURE 4:**
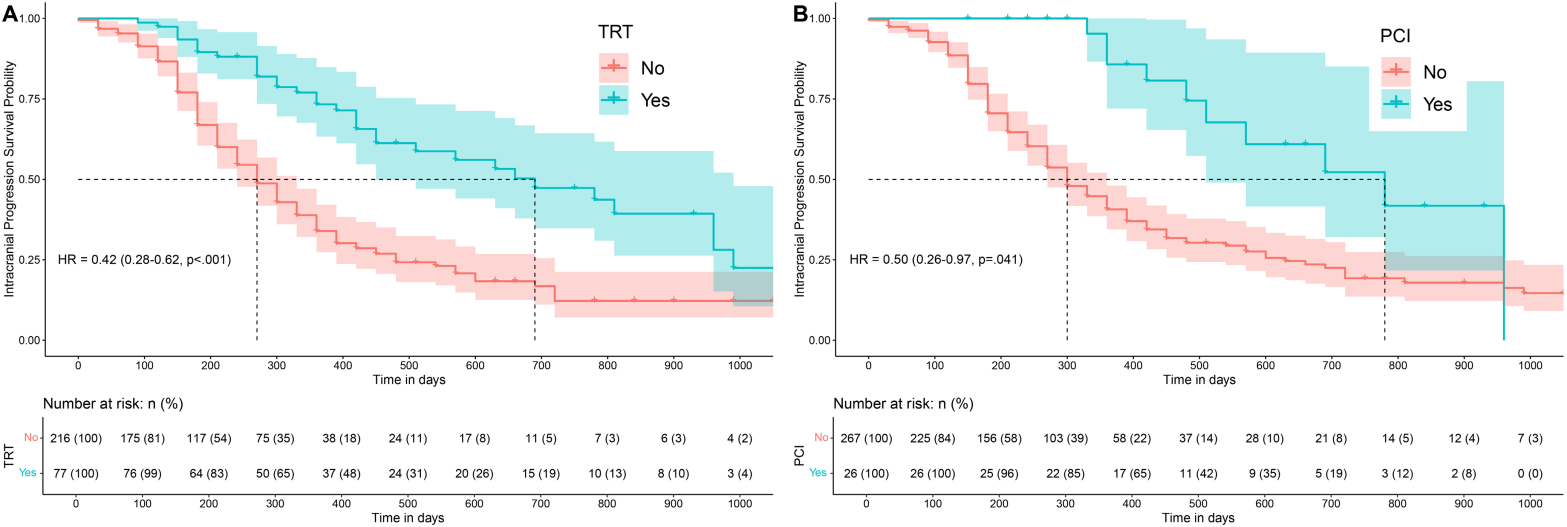
Kaplan‐Meier survival curves for patients without baseline brain metastases. (A) Thoracic radiotherapy significantly improves intracranial PFS (HR= 0.42, 95% CI: 0.28‐0.62). (B) Prophylactic cranial irradiation also shows significant effect on intracranial PFS, with a HR of 0.50 (95% CI: 0.26‐0.97).

We further analyzed factors that impact intracranial progression in patients initially free from BMs, as shown in Table 4. Our Cox regression analysis revealed that intracranial progression‐free survival (iPFS) was not influenced by the type of ICI used. However, TRT to the primary lesion and PCI to brain significantly postponed intracranial progression. Among the 293 patients without initial BMs, 77 underwent TRT, and 26 underwent PCI. The median iPFS for patients receiving TRT alongside ICIs was 22.9 months, markedly longer than the 9.0 months observed in the ICI‐only group (HR = 0.42, 95% CI: 0.28–0.62; *p* < 0.001) (Figure 4A). Similarly, for patients receiving PCI plus ICIs, the median iPFS was 26.1 months compared to 9.8 months for those on ICI alone (HR = 0.50, CI: 0.26–0.97; *p* = 0.041) (Figure 4B).

**TABLE 4:** Analysis of prognostic factors using Cox proportional hazard in patients without baseline BMs.

Variable		*N* (%)	iPFS	
			HR (univariable)	HR (multivariable)
Age	< 65	174 (59.4%)		
	≥ 65	119 (40.6%)	1.03 (0.75–1.40, *p* = 0.863)	
Sex	Female	33 (11.3%)		
	Male	260 (88.7%)	0.85 (0.54–1.33, *p* = 0.470)	
Smoking	No	57 (19.5%)		
	Yes	236 (80.5%)	0.69 (0.49–0.98, *p* = 0.038)	0.65 (0.46–0.93, *p* = 0.018)
Drinking	No	158 (53.9%)		
	Yes	135 (46.1%)	0.95 (0.70–1.29, *p* = 0.761)	
ECOG.PS	≥ 2	135 (46.1%)		
	0–1	158 (53.9%)	1.06 (0.79–1.44, *p* = 0.689)	
Liver metastases	No	224 (76.5%)		
	Yes	69 (23.5%)	1.58 (1.13–2.20, *p* = 0.007)	1.27 (0.90–1.78, *p* = 0.169)
Bone metastases	No	182 (62.1%)		
	Yes	111 (37.9%)	1.74 (1.28–2.36, *p* < 0.001)	1.34 (0.98–1.84, *p* = 0.069)
Intrapulmonary metastases	No	227 (77.5%)		
	Yes	66 (22.5%)	0.64 (0.43–0.96, *p* = 0.029)	0.69 (0.45–1.04, *p* = 0.079)
Adrenal gland metastases	No	243 (82.9%)		
	Yes	50 (17.1%)	1.06 (0.72–1.58, *p* = 0.763)	
Others metastases	No	176 (60.1%)		
	Yes	117 (39.9%)	1.02 (0.75–1.38, *p* = 0.916)	
ICIs	PD‐1	154 (52.6%)		
	PD‐L1	139 (47.4%)	1.22 (0.90–1.65, *p* = 0.203)	
TRT	No	216 (73.7%)		
	Yes	77 (26.3%)	0.39 (0.27–0.56, *p* < 0.001)	0.42 (0.28–0.62, *p* < 0.001)
PCI	No	267 (91.1%)		
	Yes	26 (8.9%)	0.34 (0.18–0.64, *p* < 0.001)	0.50 (0.26–0.97, *p* = 0.041)

Abbreviations: BMs, brain metastases; BRT, brain radiotherapy; ICIs, immune checkpoint inhibitors; PCI, prophylactic cranial irradiation; TRT, thoracic radiotherapy.

With the growing interest in determining whether PCI can be replaced in the era of immunotherapy for ES‐SCLC, we performed a subgroup analysis of PCI (Figure S2). The findings indicated that, aside from female, those with a history of smoking, those with liver or bone metastases, those receiving PD‐1 inhibitors, and those who had undergone TRT, the combination of immunotherapy and PCI showed improvement in iPFS compared to ICI. This suggests that for these specific patient groups, ICI monotherapy could be considered to avoid the cognitive impairment often associated with PCI.

## 4 Discussion

Following the IMpower133, CASPIAN, ASTRUM‐005, and CAPSTONE‐1 trials, there has been a shift toward immunotherapy combined with chemotherapy as the standard treatment protocol for ES‐SCLC [9, 10, 11, 12]. The congruence of survival outcomes in our study with extant historical data further substantiates the efficacy of immunotherapy, in which 393 ES‐SCLC patients treated with immunotherapy were included. Nevertheless, ES‐SCLC, characterized by systemic, predominantly advances to BMs [21]. Most studies have concluded that immunotherapy also improves OS and PFS in patients with BMs, similarly in our study [14, 22]. CASPIAN [22] and IMPOWER [23] trials suggested that immunotherapy and chemotherapy have been shown to delay intracranial progression. However, Lu et al. [14] underscore that immunotherapy may not suffice in curtailing the development of BMs in patients without baseline BMs. These contradictory revelations increase the exigency of investigating the efficacy of BRT combined with immunotherapy in attenuating cerebral failure rates in ES‐SCLC. There are no prospective studies evaluating the efficacy of immunotherapy on survival and intracranial control in the presence or absence of BRT. Our retrospective analysis, the most comprehensive to date, evaluates ES‐SCLC patients treated with immunotherapy and pioneers in assessing the influence of BRT combined with immunotherapy.

Radiotherapy has a synergistic effect with immunotherapy. Specifically, radiotherapy enhances antigen presentation and T‐cell activation through the release of cancer‐specific peptides, elevates the expression of major histocompatibility complex class I on tumor cells, stimulates interferon transcription, and intensifies macrophage phagocytosis [15, 24]. An alternative hypothesis posits that immunotherapy‐induced T‐cell activation may sensitize neoplasms to radiation, potentially through the normalization of tumor vasculature and alleviation of tissue hypoxia [25, 26]. For ES‐SCLC, despite typically low or negligible PD‐L1 expression in the immune microenvironment, RT can upregulate the expression of PD‐L1 on tumor cells [27]. RT may soften the BBB and increase the migration of immune cells in BMs. ICIs entering the brain can enhance the local effect of RT [28], and also enhance the systemic immune response which is manifested by the abscopal effect [28]. BRT combined with immunotherapy has been shown to have the strongest activating effect on systemic immunity in ES‐SCLC patients compared to the bone and lung radiotherapy groups, but the effect on OS was not tracked [29]. Therefore, the combination of BRT and immunotherapy plays a new role in promoting anti‐tumor response. While TRT may not directly impact the BBB, it exerts a substantial immune activation effect on pulmonary lesions. Notably, approximately 75%–90% of patients with ES‐SCLC have residual chest disease following chemotherapy, and nearly 90% experience chest disease progression within 1 year [30, 31]. Consequently, the combination of TRT and ICIs also plays a critical role in the management of ES‐SCLC patients, potentially improving outcomes by enhancing local control and augmenting systemic immune responses.

Irrespective of patients with BMs undergoing PCI, SRS, or WBRT, BRT emerges as a pivotal component in treatment strategies for them. Notably, the combination of BRT and ICIs extended OS, albeit without significantly enhancing PFS in our study. Echoing our previous findings in advanced NSCLC, no statistical difference in PFS, ORR, or DCR was observed between patients receiving ICI monotherapy and those undergoing combined BRT [32]. There are still conflicting studies [20, 33] on whether the combination of first‐line immunotherapy and BRT provides a survival benefit in ES‐SCLC patients with BMs, with one study concluding that the combination therapy did not increase the incidence of white matter lesions [33]. Our study supports that ICI plus BRT may improve OS due to the strongest immune‐activating effect of the combination [29].

To optimize the synergy of BRT and immunotherapy, identifying patients who stand to gain the most is crucial for tailoring individualized radiotherapy plans. We conducted a subgroup analysis of the baseline BMs, which indicated that patients with such characteristics, notably aged 65 and older, male, smokers, nondrinkers, with an ECOG performance status of 2 or higher, without liver or bone metastases, and with limited asymptomatic brain lesions (1–4 lesions, < 10 mm in size), ICI + BRT significantly improved OS compared to ICI. Intriguingly, a significant survival advantage was observed in patients who had not previously undergone TRT. Multiple studies [34, 35, 36] have confirmed that ICIs in combination with TRT can significantly reduce intrathoracic failure, improve OS, and delay the need for second‐line therapy, which is similar to our findings. Experts recommend that TRT be followed in patients with ES‐SCLC who have a tumor response and residual intrathoracic lesions after first‐line chemoimmunotherapy. In real life, it is quite common for patients to have a bulky disorder in the chest at the time of onset. Because patients with large tumors, interstitial lung disease, and poor lung function cannot undergo TRT, resulting in chest failure, how to improve the survival of patients is a tough question. Our study found that ICI + BRT was significantly superior to ICI in patients who had not previously received TRT. Conversely, among patients with a history of TRT, the survival advantage of combining ICI with BRT was less pronounced, potentially due to the pre‐existing influence of the immune‐activating effect. These findings highlight the critical importance of BRT + ICI for patients with BMs who have not received TRT, underlining its necessity as a treatment intervention.

Our investigation also revealed that while PCI does not enhance OS in patients without baseline BMs, it significantly extends the period before the onset of BMs. Some retrospective studies suggest that PCI and ICI can improve OS and brain metastases‐free survival, but the accuracy of the results is limited by the number of patients who undergo PCI [37, 38]. Unfortunately, there are no prospective clinical trials of ICI combined with PCI. The status of PCI may be affected by the active control of intracranial lesions by immunotherapy. In the modern era of MRI, the indications for PCI in SCLC have been questioned. MRI monitoring with salvage BRT may have greater benefits on cognitive function and quality of life than PCI. Ongoing trials such as SWOG 1827 (clinicalTrials.gov: NCT04155034) comparing PCI with MRI monitoring. Before the advent of immunotherapy, both the EORTC 22993 trial [39] which included 286 patients with ES‐SCLC, and a Japanese trial [40] that included 224 patients with ES‐SCLC reported that PCI reduced the incidence of BMs; however, the effect on OS was inconsistent. In our study, despite the absence of OS improvement, we advocate that PCI remains a relevant consideration in the immunotherapy era. Clinical decision‐making should involve a holistic evaluation of both the efficacy and toxicity of PCI, to determine its appropriate application in patient treatment plans.

The determination of an optimal sequence and interval for BRT combination with immunotherapy is a subject of substantial clinical interest and warrants further exploration. Our prior research on BMs suggests enhanced efficacy when BRT is synchronized with ICIs, particularly with minimal intervals [15]. Additionally, a sequence initiating with radiotherapy followed by immunotherapy seems more effective than the reverse order. However, in our retrospective analysis, HRs of 2.04 for PFS were observed in favor of ICI‐RT compared to RT‐ICI. This lends further credence to the importance of administering immunotherapy in ES‐SCLC. Moreover, compared to a 1‐week interval, whether 1 week to 1 month or beyond, exhibited HRs not exceeding 1. Nevertheless, no statistical significance was observed for interval, possibly attributed to the retrospective nature and limited sample size of our study, leading to potential selection bias. Additionally, the lack of comprehensive data on BRT sequencing and intervals, due to treatment across institutions, further complicated the formulation of a conclusive BRT protocol and patient selection criteria. We also delved into the comparative efficacy of SRS and WBRT. Despite no significant statistical differences observed, WBRT remained predominant, with a gradual increase in SRS utilization noted. Patients initially treated with WBRT exhibited superior intracranial control compared to those receiving SRS; however, this did not translate into an OS benefit. The reasons might be attributed to reduced neurocognitive decline associated with SRS or shorter intervals between SRS and ICIs [41]. Prospective studies are, thus, imperative to refine treatment protocols and optimize therapeutic outcomes.

Intriguingly, our research indicates that PD‐1 inhibitors may confer superior OS compared to PD‐L1 inhibitors in patients regardless of BMs, as was the case in NSCLC. This observation diverges from previous studies [42, 43] suggesting that there was no clear difference between PD‐L1 and PD‐1 inhibitors in ES‐SCLC, and one study [42] found that PD‐L1 inhibitors had a tendency to reduce the risk of death in ES‐SCLC patients with BMs compared to PD‐1 inhibitors. These studies attribute the lack of difference between PD‐L1 inhibitors and PD‐1 inhibitors to the fact that PD‐L1 is usually low or absent in SCLC [44]. Another possible explanation is that PD‐L1 binds two receptors, PD‐1 and B7.1 (CD80), and B7.1 is a key co‐stimulatory molecule on tumor‐associated dendritic cells (DC) that enhances T‐cell initiation through B7.1/CD28 interaction [45]. Although it is not possible to determine the superiority or inferiority of PD‐L1 and PD‐1 inhibitors, the effect of serplulimab has been best confirmed [46]. The potential mechanisms for the difference between PD‐L1 inhibitors and PD‐1 inhibitors in SCLC with BMs remain to be investigated by animal models.

## 5 Conclusion

Our findings underscore that radiotherapy retains its critical role in treating ES‐SCLC, even in the contemporary landscape of immunotherapy. While BRT did not demonstrate an improvement in progression survival for patients with BMs, its synergistic use with ICIs markedly enhanced OS. TRT delivers substantial survival benefits to patients with and without baseline BMs. For patients who are unable to undergo TRT, active BRT can provide a survival benefit. PCI notably extends the interval before the development of BMs in patients without baseline BMs. In clinical practice, it is imperative to consider a spectrum of patient‐specific clinical factors to devise a tailored radiotherapy regimen, optimizing treatment outcomes in ES‐SCLC.

## Author Contributions

Q.Z., X.C., Z.T., and R.Z. designed and performed the experiments, analyzed the data, and prepared the figures; G.X., J.Z., Y.Z., B.L., and L.Y. provided the key technique mentoring and research resources; Z.T. and R.Z. supervised the project; Q.Z. and X.C. wrote the manuscript. All authors read and approved the final manuscript.

## Conflicts of Interest

The authors declare no conflicts of interest.

## Transparency Statement

The design, data collection, analysis, and interpretation of the results in this study adhere to rigorous scientific standards. All relevant data and methodologies have been clearly described in this manuscript, and any reproducible data are available upon request. There has been no selective reporting, and all hypotheses were based on a prespecified analysis plan.

## Supporting information


**Figure S1.** Subgroup analysis of OS for patients with baseline brain metastases: ICI + BRT versus ICI.

**Figure S2.** Subgroup analysis of iPFS for patients with baseline brain metastases: ICI + BRT versus ICI.

**Table S1.** Patient characteristics with brain metastases.

## Data Availability

Original data are available from the corresponding author on reasonable request. The corresponding author had final responsibility for the decision to submit for publication.
