# Supplementary figures and images for: The Optimal Radiotherapy Strategy for Patients With Small Cell Lung Cancer and Brain Metastasis: A Retrospective Analysis
Source: CNS Neurosci Ther. 2024 Nov 5;30(11):e70102. doi: 10.1111/cns.70102 (PMC11537770; doi:10.1111/cns.70102)

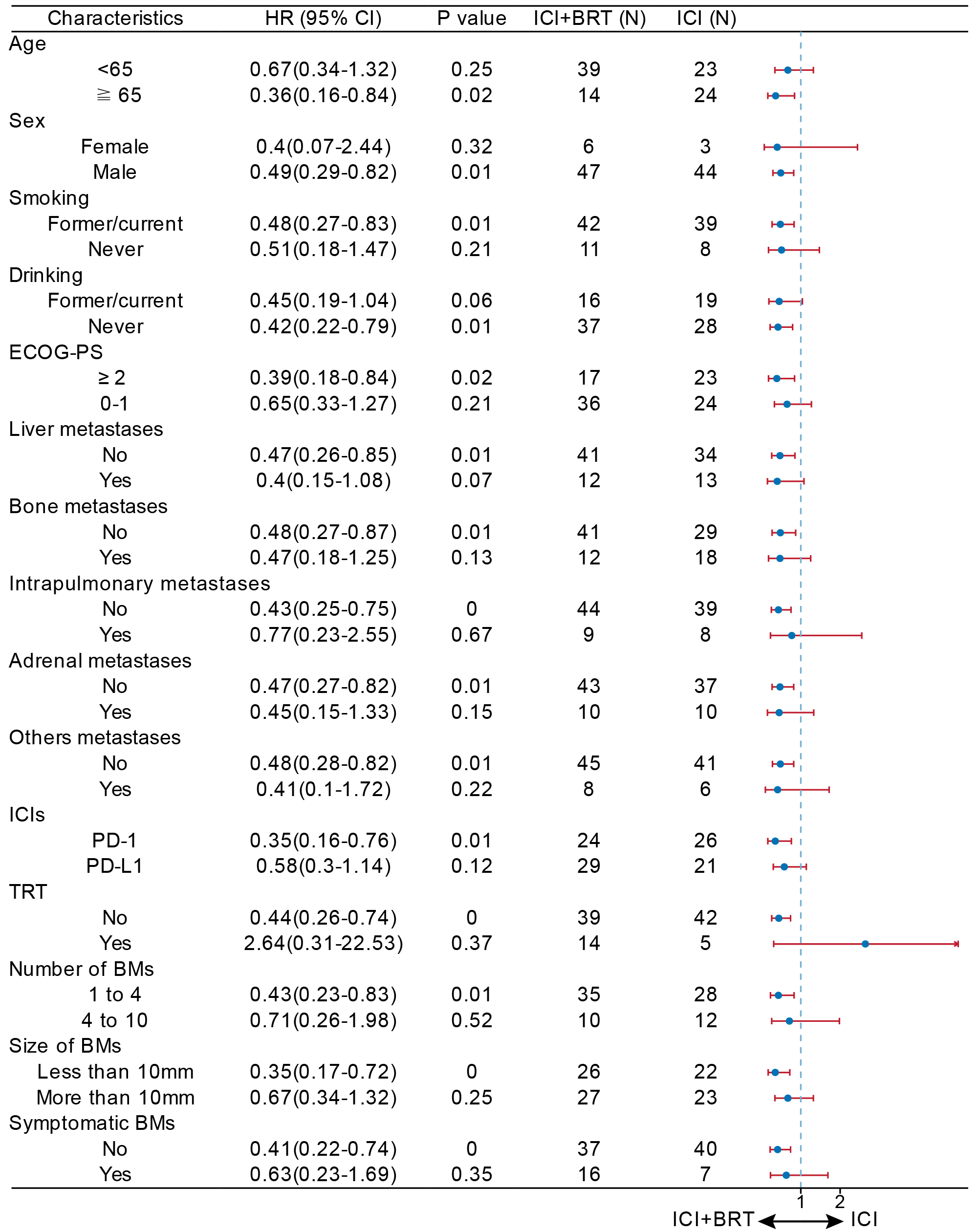

Supplement: Supplementary file 1 — Figure S1. Subgroup analysis of OS for patients with baseline brain metastases: ICI + BRT versus ICI. [file CNS-30-e70102-s002.jpg]

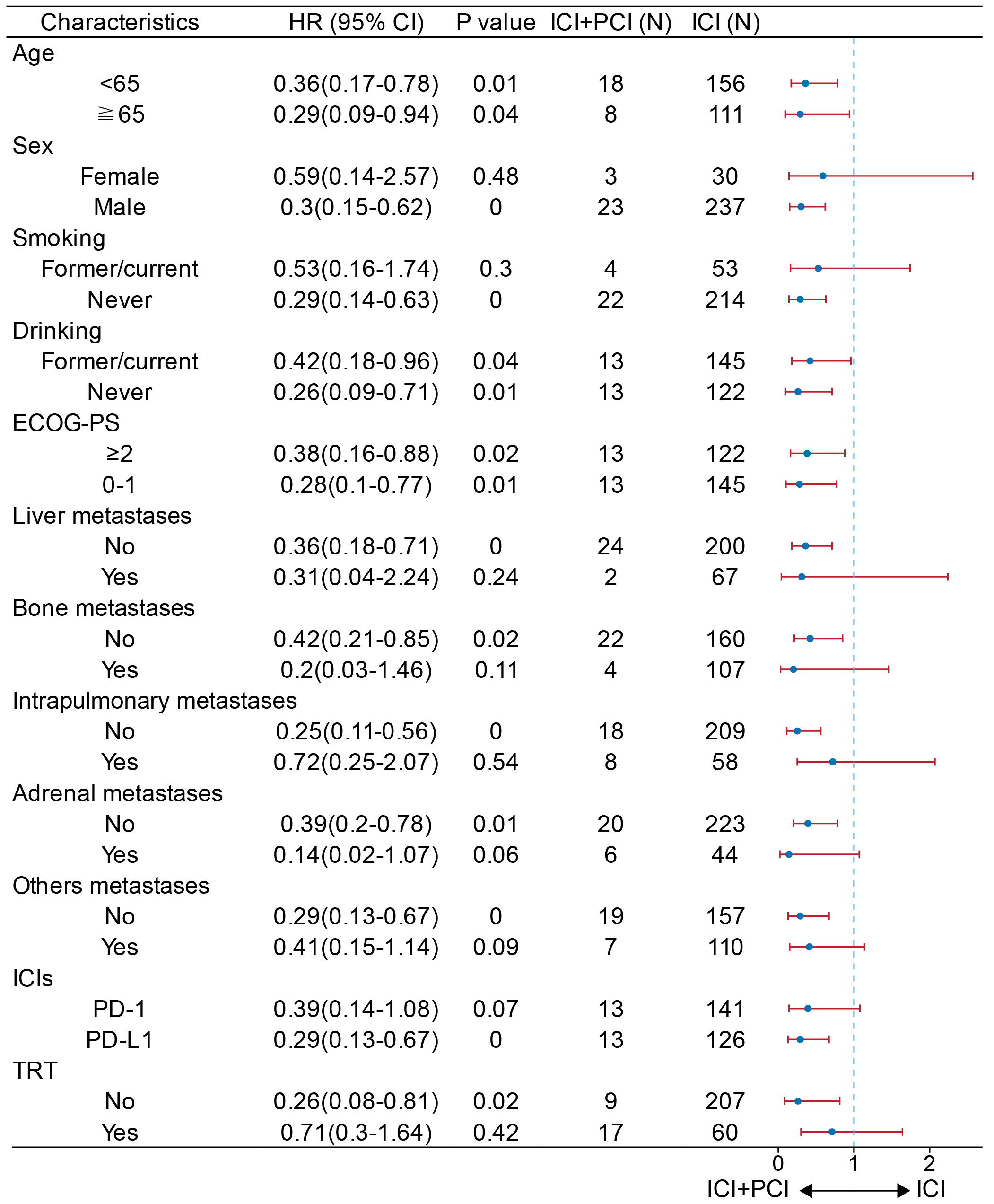

Supplement: Supplementary file 2 — Figure S2. Subgroup analysis of iPFS for patients with baseline brain metastases: ICI + BRT versus ICI. [file CNS-30-e70102-s003.jpg]
