# Supplementary material for: The Optimal Radiotherapy Strategy for Patients With Small Cell Lung Cancer and Brain Metastasis: A Retrospective Analysis
Source: CNS Neurosci Ther. 2024 Nov 5;30(11):e70102. doi: 10.1111/cns.70102 (PMC11537770; doi:10.1111/cns.70102)
Supplement: Supplementary file 3 — Table S1. Patient characteristics with brain metastases. [file CNS-30-e70102-s001.docx]

**Supplemental Table 1 Patient characteristics with brain metastases**

|  | Baseline BMs  (*N=100)* | Treatment Progression  (*N=69)* | p.value |
| --- | --- | --- | --- |
| **ICIs type, n (%)** |  |  | 0.498 |
| PD-1 | 50 (50.0%) | 30 (43.5%) |  |
| PD-L1 | 50 (50.0%) | 39 (56.5%) |  |
| **The number of BMs, n (%)^⸸^** |  |  | 0.007 |
| 1 to 4 | 69 (69.0%) | 38 (55.1%) |  |
| 4 to 10 | 20 (20.0%) | 10 (14.5%) |  |
| More than 10 | 10 (10.0%) | 14 (20.3%) |  |
| **The size of BMs, n (%)^⸸^** |  |  | 0.013 |
| Less than 10mm | 50 (50.0%) | 26 (37.7%) |  |
| More than 10mm | 49 (49.0%) | 36 (52.2%) |  |
| **Symptomatic BMs, n (%)^⸸^** |  |  | 0.106 |
| No | 76 (76.0%) | 49 (71.0%) |  |
| Yes | 24 (24.0%) | 17 (24.6%) |  |
| **Brain radiotherapy, n (%)** |  |  | 0.296 |
| No | 47 (47.0%) | 26 (37.7%) |  |
| SRS | 12 (12.0%) | 7 (10.1%) |  |
| SRS+WBRT | 4 (4.0%) | 2 (2.9%) |  |
| WBRT | 37 (37.0%) | 34 (49.3%) |  |
| **PCI, n (%)** |  |  | 0.323 |
| No | 100 (100.0%) | 67 (97.1%) |  |
| Yes | 0 (0.0%) | 2 (2.9%) |  |

Abbreviation: BMs: Brain metastases; ICIs: Immune checkpoint inhibitors; SRS: Stereotactic radiosurgery; WBRT: Whole brain radiotherapy; PCI: Prophylactic cranial irradiation.
